# Post-weaning Environmental Enrichment, But Not Chronic Maternal Isolation, Enhanced Ethanol Intake during Periadolescence and Early Adulthood

**DOI:** 10.3389/fnbeh.2016.00195

**Published:** 2016-10-13

**Authors:** Luciana R. Berardo, María C. Fabio, Ricardo M. Pautassi

**Affiliations:** ^1^Instituto de Investigación Médica Mercedes y Martín Ferreyra, Consejo Nacional de Investigaciones Científicas y Técnicas, Universidad Nacional de CórdobaCórdoba, Argentina; ^2^Facultad de Psicología, Universidad Nacional de CórdobaCórdoba, Argentina

**Keywords:** ethanol, wistar, maternal separation, environmental enrichment, adolescence

## Abstract

This study analyzed ethanol intake in male and female Wistar rats exposed to maternal separation (MS) during infancy (postnatal days 1–21, PD1–21) and environmental enrichment (EE) during adolescence (PD 21–42). Previous work revealed that MS enhances ethanol consumption during adulthood. It is still unknown if a similar effect is found during adolescence. Several studies, in turn, have revealed that EE reverses stress experiences, and reduces ethanol consumption and reinforcement; although others reported greater ethanol intake after EE. The interactive effects between these treatments upon ethanol’s effects and intake have yet to be explored. We assessed chronic ethanol intake and preference (12 two-bottle daily sessions, spread across 30 days, 1st session on PD46) in rats exposed to MS and EE. The main finding was that male – but not female – rats that had been exposed to EE consumed more ethanol than controls given standard housing, an effect that was not affected by MS. Subsequent experiments assessed several factors associated with heightened ethanol consumption in males exposed to MS and EE; namely taste aversive conditioning and hypnotic-sedative consequences of ethanol. We also measured anxiety response in the light-dark box and in the elevated plus maze tests; and exploratory patterns of novel stimuli and behaviors indicative of risk assessment and risk-taking, via a modified version of the concentric square field (CSF) test. Aversive conditioning, hypnosis and sleep time were similar in males exposed or not to EE. EE males, however, exhibited heightened exploration of novel stimuli and greater risk taking behaviors in the CSF test. It is likely that the promoting effect of EE upon ethanol intake was due to these effects upon exploratory and risk-taking behaviors.

## Introduction

Pilatti et al. (2013a) indicated lifetime prevalence of alcohol sipping or tasting in 50% (females) to 70% (males) of Argentinean children aged 8–12 years-old. Lifetime prevalence of alcohol drinking (i.e., ≥1 full drink) was 34.3%. Another study, conducted in the same city and in an older sample (mean age = 20 years) of similar sociodemographic characteristics, indicated less than 7% of abstainers, with most of the subjects reporting last month drinking and approximately half of them reporting an average of 4/5 drinks per drinking occasion, which constitutes binge drinking associated with several adverse consequences, including greater likelihood of alcohol abuse and dependence (Gruber et al., 1996; DeWit et al., 2000; Pilatti et al., 2013b). Together, these studies illustrate the pathway from initiation to sustained alcohol use that, almost normatively across cultures, takes place during late infancy and adolescence.

Epidemiological and animal research has indicated that the quality of the early (maternal and then fraternal/peer) environment is a key factor to accelerate or deter from alcohol engagement during infancy and adolescence. Subjects who experienced early life stress are more likely to begin drinking early in life (Rothman et al., 2008; Enoch, 2012) and to report stress coping as a motive for drinking during the first year of drinking (Rothman et al., 2008). Early onset of drinking, in turn, increases the risk for stress-related drinking (Dawson et al., 2007) and predicts subsequent alcohol abuse and dependence (DeWit et al., 2000). Conversely, social enrichment during adolescence reverses the social deficits observed in rats exposed to ethanol (Middleton et al., 2012) or valproic acid (Schneider et al., 2006) during pregnancy. The effects of early life environmental conditions on reactivity to ethanol can be assessed via the maternal separation (MS) (Francis and Kuhar, 2008) or the environmental enrichment (EE) experimental preparations (Rueda et al., 2012).

In the MS preparation, rats experience 180 or 360 min of maternal separation (commonly referred to as MS180 or MS360 treatments, respectively), every day from postnatal day (PD) 1 to PD14 or until weaning on PD21 (Kawakami et al., 2007). Maternally separated animals exhibit, when tested at adulthood, enhanced ethanol self-administration and greater hormonal and behavioral responsiveness to stress (Huot et al., 2001; Cruz et al., 2008) than animals reared under normal animal facility rearing (AFR) conditions. The home cage of rodents exposed to EE features several combinations of interactive objects, including tunnels, toys and running wheels that provide opportunity for voluntary physical activity. EE holds promise as a non-pharmacological alternative to reduce ethanol-induced reinforcement and intake. Exposure to EE inhibits ethanol consumption and reduces the magnitude of ethanol- (de Carvalho et al., 2010) or cocaine-induced (Solinas et al., 2009) conditioned place preference in rats. Moreover, adolescent mice exposed to EE were insensitive to the increase in motor stimulation observed after repeated and intermittent ethanol administration (i.e., ethanol-induced behavioral sensitization) (Rueda et al., 2012).

Few animal studies assessed the effects of maternal separation soon after termination of this treatment, during infancy or adolescence. These studies have reported no effect of MS upon ethanol drinking at adolescence or periadolescence, although alterations in open field activity and play behavior were observed (Arnold and Siviy, 2002). Daoura et al. (2011) found greater ethanol intake in MS360 vs. AFR animals when testing began at adulthood, but not when testing began at adolescence; whereas others (Palm et al., 2013) found no differences in ethanol consumption in adolescent, male Wistar rats, subjected to MS360 or control conditions. The effects of EE upon ethanol drinking during adolescence have not been explored. Moreover, although most of the available suggests that EE may reduce ethanol-seeking (Roman et al., 2003; Daoura et al., 2011), there are contradictory results. Long-term exposure to EE (i.e., 3 or more months) was associated with significantly greater ethanol intake in adults, genetically heterogeneous rats (Rockman et al., 1989) and in rats selected for low or high anxiety response (Fernández-Teruel et al., 2002). It is still an open question whether the promoting effects of MS upon ethanol intake are immediately evident during adolescence or whether they follow a more delayed pattern of expression, appearing only later in development, after brain maturation. Also unknown is if EE will serve as potential treatment to reduce ethanol engagement during adolescence. The interactive effects between these treatments have not yet been explored.

The present study assessed, in Wistar male and female rats, the effects of maternal separation during infancy, followed by exposure to EE throughout adolescence, on ethanol drinking during periadolescence [i.e., between PD42 and PD60, Spear, 2000] and early adulthood [i.e., between PD61 and PD72]. After establishing that EE actually enhanced male ethanol drinking (Experiment 1), subsequent experiments assessed several effects of EE likely to underlie this promoting effect. We assessed EE effects, an MS modulation, of anxiety response in an elevated plus maze (EPM), aversive effects of ethanol and sensitivity to the sedative and sleep-inducing effects of ethanol (Experiment 2). Greater anxiety may facilitate ingestion of ethanol due to the anxiolytic effects of this drug (Spanagel et al., 1995), whereas the aversive and sedative effects of ethanol serve as barriers precluding further drug seeking and taking (Spear and Varlinskaya, 2010). Treatments that ameliorate these effects may promote ethanol drinking. Experiment 3 tested the hypothesis that EE may increase ethanol drinking by exacerbating the proclivity to take risks and explore new environments.

## General Materials and Methods

### Subjects

One hundred and twenty-one Wistar rats were used. Number of animals in each experiment was as follows: Experiment 1, 32 males, 32 females (derived from 8 l, four experienced AFR, four experienced daily episodes of MS); Experiment 2, 32 males (derived from 8 l, four experienced AFR, four experienced daily episodes of MS); and Experiment 3, 32 males (derived from 8 l, four experienced AFR, four experienced daily episodes of MS). These animals were born and reared at the production vivarium at INIMEC-CONICET-Universidad Nacional de Córdoba (Córdoba, Argentina), which is kept at 22–24°C with a12 h/12 h light/dark cycle (lights on at 8:00 AM). The pregnant dams came from the regular stock of the vivarium and births were checked daily. The day of birth was considered as PD0 and on PD1 litters were culled to four females and four males. Subjects were naïve to experimental procedures in each Experiment. Unless specified, litters were housed in standard maternity cages and given *ad libitum* access to water and lab chow. The experimental protocol was reviewed and approved by Institutional Animal Care and Use Committee (CICUAL protocol No. 2014-10) and complied with the regulations of the Guide for Care and Use of Laboratory Animals (National-Research-Council, 1996).

Litter effects across experiments were controlled by including no more than one male or female per litter in any group condition and by conducting a cross-fostering procedure at PD1, after the culling and before commencement of experimental treatment. More in detail, at PD1 the dams were briefly moved to a separate, clean cage and two males and two females from a given litter were transferred to another litter, which in turn provided two males and two females to the former litter. This procedure helped avoid assigning more than one male or female from the same litter to a given experimental group. Specifically, any given litter was randomly assigned to the MS or AFR condition. Within each litter, two males (one fostered and one not fostered) were assigned (at weaning time) to the EE condition and 2 were assigned the CTRL condition. The same procedure was done for females. Had we chosen not to conduct cross-foster, we would have needed additional litters, because we could have only assigned one male and one female to the post-weaning EE and CTRL conditions. Informal observations of the dam’s behavior upon their return to the homecage indicated that the pups were immediately accepted by the foster dams, which readily exhibited a normal maternal behavioral repertoire (e.g., pup retrieval, nest building, licking, and grooming of the pups).

### Rearing Conditions across PDs 1–21 (Experiments 1, 2, and 3)

**FIGURE 1:**
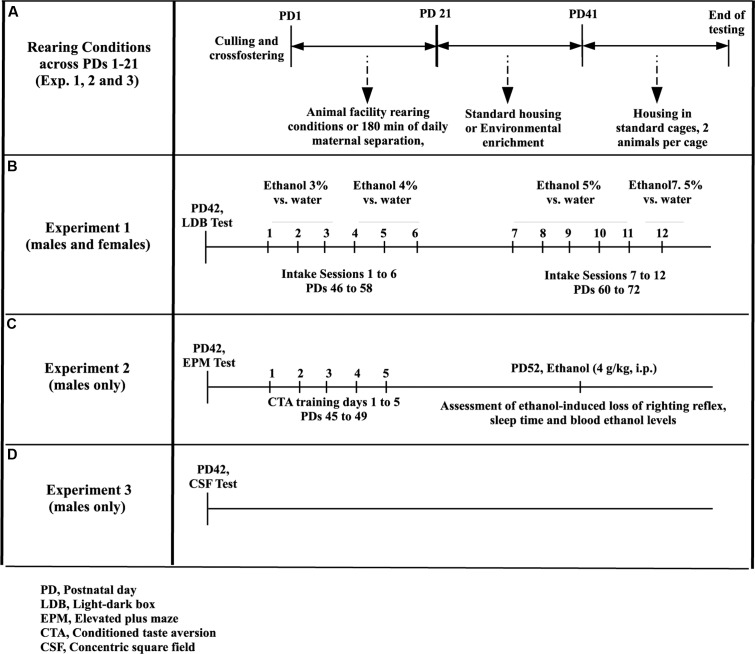
**Methods for the analysis of the effects of maternal isolation during infancy and environmental enrichment during adolescence on ethanol intake in adolescent Wistar rats.** **(A)** From postnatal day (PD) 1 to PD21, the rats were reared under animal facility rearing conditions or were given daily episodes of maternal isolation (180 min). Between PD21 and PD42 they were given standard housing or environmental enrichment. The tests conducted on Experiments 1, 2, and 3 began on or after PD42. **(B)** The rats (males and females) were assessed for ethanol intake (Experiment 1) during 4 weeks, from PD46 to PD72. During each week animals were given three every other day, two-bottle choice tests (ethanol vs. plain water), followed by two rest days. Ethanol concentration was 3% (week 1, intake sessions 1–3), 4% (week 2, intake sessions 4–6), 5% (weeks 3 and 4, intake sessions 7–11) or 7.5% (session 12). **(C)** The rats, males only, were tested in an elevated plus-maze test at PD42. Taste conditioning, employing ethanol (2.5 g/kg, i.p.) as the unconditional stimulus, was acquired on PD47 and tested on PD49. Animals were given 4.0 g/kg ethanol (i.p.) and tested for ethanol-induced loss of righting reflex, sleep time and blood ethanol levels at PD52. **(D)** In Experiment 3, male rats exposed or not to MS during infancy and reared under EE or controls conditions during adolescence were tested on PD42, in a modified version of the concentric square field (CSF).

On PD 1, litters were randomly assigned to the AFR condition or to experience 180 (Experiments 1, 2m and 3, see **Figure 1**) min of daily MS, once daily during PD 1–21. MS followed a standardized protocol, commonly used in our lab (see Fernandez et al., 2014). At 0900 AM the pups were removed from the dam and placed, as a litter, in a room located next to the housing room, in a clean maternity cage. The cage was equipped with a heating pad that kept floor temperature at 35°C. The pups were returned with the dam at noon. The dam stayed in the homecage during the MS procedure. AFR and MS litters were exposed to a weekly change in maternal cages and beddings.

### Rearing Conditions across PDs 21–42 (Experiments 1, 2, and 3)

**FIGURE 2:**
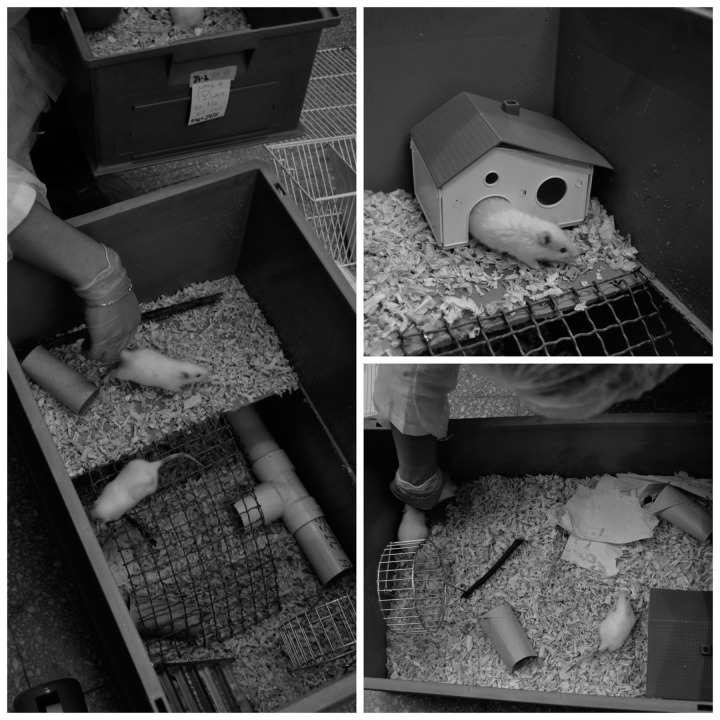
**Wistar rats under environmental enrichment conditions were housed in large opaque cages (60 cm length × 40 cm width × 40 cm height) that contained seven objects and toys, including ladders, cylinders, pipes, house-like objects, and a running wheel.** The photograph illustrates these conditions.

After termination of the maternal separation session on PD21 (weaning day in most rodent breeding protocols), the animals were randomly assigned to EE or standard (control) housing (CTRL). Control rats were transferred, in same-sex groups of four, to a standard cage (60 cm length × 40 cm width × 20 cm height), and rats in the EE groups were housed in same-sex groups of four in similar, yet taller, cages (60 cm length × 40 cm width × 40 cm height) that featured two levels connected by a ramp and equipped with seven objects and toys, including ladders, cylinders, pipes, house-like objects, and a running wheel. Food was placed always in the floor, in a corner. To prevent habituation, the experimenter changed the location and composition of the objects twice a week. **Figure 2** illustrates one of these compositions. The animals were kept under EE or CTRL conditions until the morning of PD42. This is, EE was conducted throughout the juvenile and adolescent stages of development. Following recommendations from our institutional animal care committee the rats were pair-housed in same-sex couples after PD42. This recommendation takes into account the relationship between size of the homecage and weight of the animal. A succinct description of the rearing protocol can be found in **Figure 1.**

### Light-Dark Box (LDB, Experiment 1) Test

In Experiment 1, animals were tested in an LDB apparatus at PD42, immediately after termination of EE exposure. This day the animals were withdrawn from the home-cage, which was still enriched for those in EE groups. The LDB featured two compartments made of high impact acrylic, one white (24.5 cm × 25 cm × 25 cm) illuminated by a 60 W white bulb lamp adjusted to generate an illumination level of 400 lux, and one black (17.5 cm × 25 cm × 25 cm) without illumination (i.e., 0 lux). A divider with an opening at floor level separated both compartments. The test began by gently placing the animal in the center of the white area, facing away from the black area. After termination of the 5-min LDB test, all animals were housed in standard cages (two animals per cage). Illumination of the apparatus was being measured via a digital lux meter (LX1010B). The following variables were measured: number of transfers between compartments, latency (s) to enter the dark compartment, time (s) spent in the white compartment and frequency of stretching behavior.

### Ethanol Intake Procedures (Experiment 1)

We used a two-bottle intake procedure, described in Fabio et al. (2015), to assess ethanol intake and preference from PD46 to PD72. This period encompass periadolescence (i.e., between PD42 and PD60, Spear, 2000) and early adulthood (i.e., between PD61 and PD72). The animals went through a 4 weeks, intermittent-access ethanol intake protocol (three sessions per week starting on Monday, Wednesday, and Friday, 24 h per session; described in **Figure 1**). They were exposed to 3, 4, and 5% ethanol vs. plain water, on weeks 1–3, respectively. Animals were also exposed to 5% ethanol vs. plain water in the Monday and Wednesday sessions of week 4. On the last test session on Friday, however, the animals were “challenged” with 7.5% ethanol vs. plain water.

Animals were housed individually during the course of each 24 h test. Before and after each intake session, however, the animals were pair-housed in same-sex couples with *ad libitum* access to food and water. More in detail, during intake sessions, each animal was individually housed in half (i.e., 27 cm × 18.5 cm × 20 cm) of a standard homecage and separated from its conspecific by a divider made of high impact acrylic (27.5 cm × 18.5 cm). Each half of the homecage had a metal lid that accommodated food pellets and two bottles. The animals and the bottles were weighed before and after each session. These records were used to calculate ethanol intake on a gram per kilogram (g/kg) basis, and percent (%) selection of ethanol intake. Leakage was accounted for by having a bottle of ethanol and a bottle of ethanol in an empty cage, located next to the experimental cages. The readings of these bottles were subtracted from the amount of the corresponding fluid (ethanol, vehicle) registered in each cage.

### Elevated-Plus Maze (EPM) Test (Experiment 2)

Experiment 1 indicated an effect of EE on ethanol intake, in males only. Experiment 2 was aimed at analyzing mechanisms underlying this effect of EE and employed only male rats. These males were exposed to MS or AFR during infancy and reared under EE or CTRL conditions during adolescence. They were submitted to a 5-min EPM test on PD42, immediately after termination of EE or CTRL housing, and before commencement of standard housing.

The EPM was made of black metal with a black Plexiglas cover and consisted of two open, unprotected arms (45 cm × 5 cm) and two closed, protected arms (45 cm length × 5 cm width × 45 cm height) that extended from a central platform (5 cm × 5 cm) elevated 50 cm above the floor. Each rat was placed in the central platform facing an open arm. Percentage of entries into the open arms and total number of arm entries were calculated and considered an index of anxiety response and overall exploratory behavior, respectively. The following naturalistic behaviors were recorded as well: rearing (standing on the hind limbs not in contact with a wall), stretching (propelling the body forward while keeping immovable the hind paws), sniffing (head upward with movement of the nostrils), and head-dipping (positioning the head out of the maze border and below the floor level). Grooming, defined as strokes over the nose that were eventually followed by large bilateral strokes and body licking (Arias et al., 2010), was not observed. Due to their definition, rearing and head dipping can only be performed in the open arm. These behaviors are exploratory behaviors associated with exploration of novelty (Fernández-Teruel et al., 2002; Lever et al., 2006). Stretching, indicate of risk assessment (Bailey and Crawley, 2009) was measured toward the open and closed arms (no stretching was observed toward the center area), whereas sniffing was measured in the open and closed arms, and in the center section.

### Taste Aversive Conditioning (Experiment 2)

The rats were submitted to a 5-day taste aversion conditioning protocol, which began 3 days after the EPM test (see **Figure 1**). The aim was to analyze potential EE-induced modulation of the aversive effects of ethanol, which are key regulators of ethanol intake (Dyr et al., 2016). The procedure has been commonly used in our lab (Acevedo et al., 2010; Fabio et al., 2015). On PD45 (day 1), the adolescents were housed individually and given *ad libitum* access to food and water. On the morning of the next day (PD46) the bottle of water was replaced by a new bottle filled with 50% of the volume of water they had drank during the previous day. On day 3 (PD47), the animals were weighed to the nearest 0.1 g. Upon returning to the cage the water bottle had been substituted by a graded tube containing a 0.09% sodium chloride solution. Animals had free access to the solution for 30-min, then sodium chloride intake was measured and animals were immediately administered ethanol (2.5 g/kg, i.p., concentration: 21%, mixed in physiological saline, volume of administration: 0.015 ml per gram of body weight). On day 4 (PD48), the adolescents were again given 50% of the volume of water they had ingested on day 1 (corrected by the weight registered on PD48). Aversive conditioning was assessed on day 5 (PD49). On the morning of that day the water bottle was replaced by a graded tube containing a 0.09% sodium chloride solution. Intake was recorded after 30 min and expressed as milliliters consumed per 100 g of the rat (ml/100 g).

### Assessment of Ethanol-Induced Loss of Righting Reflex, Sleep Time and Blood Ethanol Levels (Experiment 2)

Ethanol’s sedative and sleep-inducing effects limit sustained engagement in ethanol self-administration (Spear and Swartzwelder, 2014). Experiment 2 assessed these effects 3 days after termination of the taste aversive conditioning (see **Figure 1**). All animals had been administered ethanol during the aversive conditioning, thus they were equated in terms of ethanol exposure when the test for ethanol-induced sleep began.

On PD52 the rats, were i.p., injected with ethanol (4.0 g/kg, concentration: 21%, vehicle: physiological saline, volume of administration: 0.024 ml per gram of body weight) and immediately monitored. Signs of sedations lead the experimenter to position the animal in a supine position. If the animal turned over the experimenter would put him back again in a supine position. The loss of the righting reflex was considered when the animal was not able to recuperate the prone posture three times in 30 s. The period elapsing between times of loss to time of regaining the righting reflex was considered sleep time. The animal that regained the prone posture when placed supine three times within a 30 s interval was considered recovered.

Blood trunk (2 ml) samples were obtained at recuperation through decapitation, using a capillary tube with heparin. The samples were kept at -70°C for later analysis of blood ethanol concentrations, via a Hewlett-Packard gas chromatographer (Model 5890). The vials containing the samples were incubated into a hot water bath (60°C) for 30 min and then a gas-tight syringe (Hamilton Co., Reno, NV, USA) was used to extract the volatile component of each vial, which was in turn injected into the chromatographer. The carrier gas was nitrogen (speed: 15 ml/min) and the column, oven and detector were set at 60, 150, and 250°C, respectively.

### Assessment of Shelter Seeking, Exploratory and Risk Taking Behaviors (Experiment 3)

In Experiment 3, male rats exposed to AFR or MS during infancy and reared under EE or CTRL conditions during adolescence were tested (PD42) in a modified version of the concentric square field (CSF, first described by Meyerson et al., 2006). The CSF, which is usually used in adult rats (Karlsson and Roman, 2016), features a central square interconnected to several other areas by corridors. Some of the areas evoke shelter-seeking behavior, whereas others evoke exploration, risk assessment and risk taking. The front of the maze is a high-risk, brightly illuminated area with an elevated wire mesh structure that animals can climb. Compared with other tests, the CSF allows simultaneous measurement of different behavioral patterns, allowing investigating a broader behavioral profile (Roman et al., 2012). The CSF does not impose subjects a single or binary behavioral option but instead allows a graded set of exploratory activities that bridge the gap from seeking sheltered, enclosed dark spaces to seeking illuminated and elevated spaces that entail potential high-risk (Karlsson and Roman, 2016).

The CSF (48 cm × 48 cm) was made of black melamine, except for the front side wall (i.e., next to the bridge), which was made of transparent PET. The external walls were 48 cm high and the internal walls were 40 cm high. The central square (26 cm × 26 cm) gave access to three corridors (A, B, C). Corridor A led to the dark shelter (SHEL, 10 cm × 15 cm × 40 cm), which was the only enclosed section of the maze. Corridor B (18 cm × 10 cm × 48 cm) led to the challenge (CHA) area, so called because animals had to jump through a hole, elevated 10 cm from the floor, to get into it. There were two of these holes in the CHA area, one led to corridor B and the other headed to corridor C (15 cm × 10 cm). The latter corridor also allowed access to the front section of the maze, a brightly illuminated runway separated from the outside by a transparent plastic. An animal coming from the C corridor to the front area first encountered a ramp (RAMP, 12 cm × 10 cm, inclination: 20°), which led to an elevated bridge (BRIDGE, 30 cm × 10 cm). RAMP and BRIDGE were made of a hard wire mesh. Lighting conditions (lx) in the CSF arena, which were established following previous studies (Karlsson and Roman, 2016) and measured by digital luxometer (LX1010B), were as follows: SHEL: 0; CF, corridors A, B, C and CHA: 20–30; RAMP and BRIDGE: 600–650. The test lasted 20 min and was video recorded for subsequent processing via ETHOLOG 2.2 (Ottoni, 2000). Time spent and frequency of entries in each section was measured, along with frequency of nose-poking in the CHA holes.

### Experimental Designs and Statistical Analysis

Experiment 1 employed a 2 (Rearing conditions during infancy: AFR or MS) × 2 (Rearing conditions during adolescence: CTRL or EE) × 2 (sex: male or female) factorial design, with eight animals per group. Animals were exposed to MS on PDs 1-21 and to EE on PDs 21–42. Anxiety responses in the LDB test (latency to exit the bright compartment, time spent in the bright compartment and number of transfer between compartments) were separately analyzed via factorial analyses of variance (ANOVAs). The dependent variables of the ethanol intake assessments [overall fluid intake (ml/100 g), and ethanol intake (g/kg and percent preference)] were examined using separate four-way mixed ANOVAs. Rearing conditions during infancy and adolescence, and Sex were the between-group factors, and Session (sessions 1–12) was the repeated measure (RM).

In Experiments 2 and 3 the rats (only males) were distributed into four groups (*n* = 8) as a function of Rearing conditions during infancy and Rearing conditions during adolescence. The anxiety responses registered during the EPM test (latency to exit from and time spent in the bright area, number of transfers between compartments) were analyzed via independent factorial ANOVAS (between factors: Rearing conditions during infancy and during adolescence). Similar ANOVAs were used to analyze latency to lose the righting reflex, ethanol-induced sleep time, blood ethanol levels at awakening time (Experiment 2) and the time spent and total number of entries in the different sections of the CSF (Experiment 3). Consumption of sodium chloride (NaCl, ml/100 of body weight) during conditioning and testing of Experiment 2 was analyzed with a 3-way RM ANOVA, in which Rearing conditions during adolescence and adulthood served as between factors and Days of assessment as RM. A significant reduction in NaCl intake between conditioning and testing was taken as an indication of taste avoidance.

The total number of section entries is a measure of exploratory activity in the CSF, but it conflates locomotion in protected and unprotected sections of the CSF. To better understand the difference in exploration of risk areas vs. exploration of sheltered/protected areas of the apparatus, the total number of entries was split between (a) entries in risk taking/assessment areas (RAMP, CHA and BRIDGE), (b) entries in the sheltered area and in the corridor A that leads to it, and (c) entries in corridors B and C. Separate RM ANOVAs (between factors: Rearing conditions during infancy and during adolescence, within factor: Section of the apparatus) were conducted for each group of variables. Separate factorial ANOVAs analyzed time spent and frequency of entries in the central sector. Another factorial ANOVAs was used to analyze nose-poking in the CHA sector.

Significant main effects and significant interactions were scrutinized via follow-up ANOVAs, *post hoc* tests or planned comparisons. More in detail, Tukey’s tests were used to scrutinize simple main effects or interaction involving “between” factors, whereas significant interactions involving RMs were analyzed through orthogonal planned comparisons. The rationale was that there is no unambiguous choice of pertinent error terms for *post hoc* comparisons involving between-by-within factors (Winer et al., 1991). The partial eta square ($\eta_{p}^{2}$) was used to estimate effect size and the alpha level was ≤0.05. STATISTICA 8.0 (StatSoft, Tulsa, OK, USA) was used for the respective statistical analyses. Data from 7 animals (3 in Experiment 1 and 4 in Experiment 3) were lost due to errors during the experimental procedures or the processing of the videotapes. These data were not replaced.

## Results

### Experiment 1

**Table 1** presents the data yielded by the LDB test, which was conducted at termination of the EE treatment and before the ethanol intake tests. Latency to exit the bright compartment was not affected by Sex or Rearing conditions, whereas the ANOVAs for number of transfers between compartments and for time spent in the bright compartment yielded significant main effects of Sex [*F*_(1,53)_ = 5.07, *p* ≤ 0.05, $\eta_{p}^{2}$ = 0.08; *F*_(1_,_53)_ = 4.13, *p* ≤ 0.05, $\eta_{p}^{2}$ = 0.07; respectively] and a significant interaction between Rearing conditions at infancy and at adolescence [*F*_(1,53)_ = 4.14, *p* ≤ 0.05, $\eta_{p}^{2}$ = 0.07; *F*_(1,53)_ = 4.91, *p* ≤ 0.05, $\eta_{p}^{2}$ = 0.08; respectively]. Females, irrespective of rearing conditions, exhibited significantly more transfers and spent significantly more time in the bright compartment than males did. The *post hoc* indicated significantly greater number of transfers in the MS-EE group than in the MS-CTRL group (*p* ≤ 0.05). The *post hoc* also revealed significantly greater time spent in the bright compartment in the MS-EE group than in groups MS-CTRL or AFR-EE (*p* ≤ 0.05). Stretching behaviors were significantly greater in animals exposed to MS than in AFR controls, an effect that was independent of rearing conditions during adolescence [significant main effect of rearing conditions during infancy: *F*_(1,53)_ = 5.60, *p* ≤ 0.05, $\eta_{p}^{2}$ = 0.10].

**Table 1:** Data (expressed as mean ± SEM) gathered in the light-dark box (LDB, Experiment 1) and elevated plus maze (EPM, Experiment 2) tests, and in the test for sensibility to the sedative and hypnotic effects of ethanol (Experiment 2).

	Males				Females			
		
	Environmental enrichment (EE) groups		Standard (Control, CTRL) housing groups		Environmental enrichment (EE) groups		Standard (Control, CTRL) housing groups	
				
LDB	Maternal separation (MS)	AFR	Maternal separation (MS)	AFR	Maternal separation (MS)	AFR	Maternal separation (MS)	AFR
Latency to exit the bright sector (s)	28.10 ± 7.42	28.10 ± 7.42	29.76 ± 18.90	10.41 ± 3.64	17.73 ± 5.88	37.29 ± 16.16	28.99 ± 12.38	28.00 ± 6.2
Transfers between sectors	2.43 ± 0.36	2.43 ± 0.36	1.25 ± 0.62	2.12 ± 0.51	4.75 ± 0.82 (#)	2.28 ± 0.75 (#)	2.62 ± 0.59 (#)	2.5 ± 0.57 (#)
Time in bright sector (s)	2.42 ± 0.37 (&)	2.42 ± 0.37	1.25 ± 0.62	2.62 ± 0.59	4.75 ± 0.82 (&) (#)	2.28 ± 0.74 (#)	1.25 ± 0.68 (#)	2.5 ± 0.57 (#)
Stretching (frequency)	1.86 ± 0.63 (^∗^)	1.86 ± 0.63	1.87 ± 0.35 (^∗^)	0.5 ± 0.27	2.12 ± 0.77 (^∗^)	2.43 ± 0.48	1.5 ± 0.60 (^∗^)	1.62 ± 0.98

**EPM**								
				
Stretching(open, closed arms)	0.63 ± 0.26,3.88 ± 0.30	2.63 ± 0.68 (&),2.75 ± 0.70	1.13 ± 0.58,3.13 ± 0.50	0.75 ± 0.25, 3.50 ± 0.87
				
Sniffing(center, open, closed arms)	3.62 ± 0.78, 4.87+/1.14, 13.00 ± 1.85 (^∗^)	2.87 ± 0.61, 4.13+/1.11, 9.25 ± 1.77	1.5 ± 0.65, 2.75+/1.21, 14.88 ± 2.75 (^∗^)	8.38 ± 2.51 4.00+/1.49, 8.38 ± 2.51
				
**Assessment of sedative effects of ethanol and BELs**				
				
Sleep time (s)	8845.34 ± 736.44	9223.57 ± 835.85	8327.96 ± 514.17	9189.50 ± 173.91
Loss of righting reflex (s)	916.83 ± 596.99	166.00 ± 9.18	144.6 ± 9.57	213.90 ± 21.07
Blood ethanol levels (mg%)	360.46 ± 30.56	415.32 ± 20.01	308.42 ± 30.83	358.65 ± 30.73


*The animals, male or female Wistar rats, were exposed or not to maternal separation on PDs 1–21 and housed under environmental enrichment or standard (control) housing conditions on PDs 21–42. Tests were conducted at PD42 (EPM, LDB) or PD52 (ethanol-induced sedation and hypnosis). The pound sign (#) indicates a significant main effect of Sex on the number of transfers between sectors and on the time spent in the bright sector (LDB test). The asterisk sign (^∗^) indicates a significant main effect of Rearing conditions during infancy on frequency of stretching (LDB test) and on sniffing (closed arms, EPM test). The ampersand sign (&) indicates that AFR-EE animals made significantly more stretching in the open arms (EPM test) than the rest of the groups.*

**FIGURE 3:**
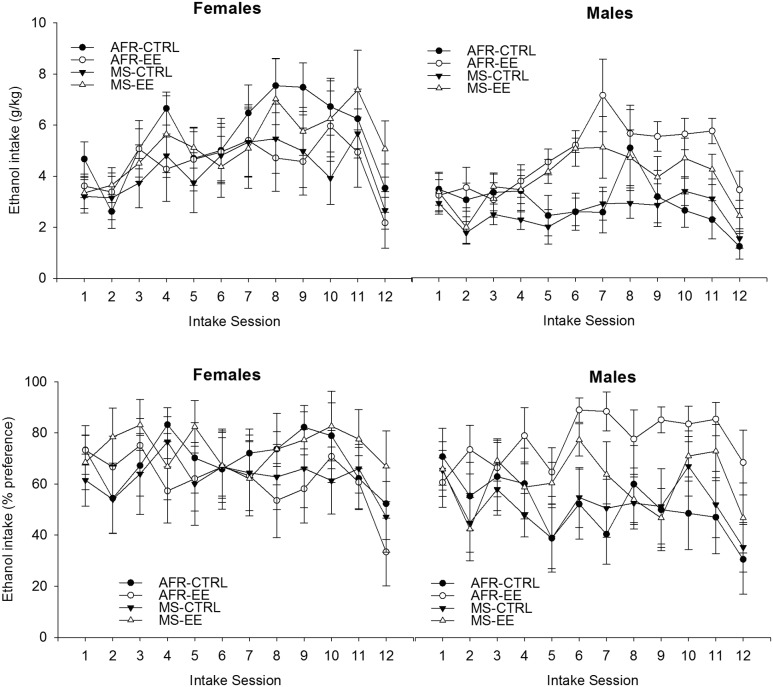
**Ethanol intake (g/kg and percent preference) (**upper and lower panels**, respectively) in male and female Wistar rats as a function of rearing conditions during postnatal days 1–21 (animal facility rearing or daily episodes of maternal separation, AFR and MS groups, respectively), rearing conditions during postnatal days 21 to 42 (standard control housing or environmental enrichment, CTRL and EE groups, respectively) and intake session.** Two-bottle intake sessions (ethanol vs. plain water) were conducted on Monday, Wednesday and Friday (session length: 24 h), during 4 weeks. Ethanol concentration was 3% (week 1, intake sessions 1–3), 4% (week 2, intake sessions 4–6), 5% (weeks 3 and 4, intake sessions 7–11) or 7.5% (session 12). The analyses of variance (ANOVAs) indicated, for both variables, a significant interaction between Sex, Rearing conditions at adolescence and Sessions. These significant interactions are depicted in **Figure 4.** Follow-up ANOVAs (Rearing conditions at adolescence × Sessions) for each sex indicated a lack of significant main effects or significant interactions in the females; whereas the ANOVAs for males indicated greater ethanol intake and percent preference in subjects given EE than in controls at sessions 5, 6, 7, 9, 10, 11, and 12 (g/kg) or 5, 6, 7, 11, and 12 (% preference). The data are expressed as mean ± SEM.

**Figure 3** illustrates ethanol intake patterns across groups. The analysis for absolute (g/kg) ethanol intake revealed significant main effects of Sex and Session [*F*_(1,53)_ = 7.05, *p* ≤ 0.05, $\eta_{p}^{2}$ = 0.12; *F*_(11,583)_ = 12.81, *p* ≤ 0.001, $\eta_{p}^{2}$ = 0.19; respectively]. The interaction between Sex, Rearing conditions at adolescence and Sessions achieved significance [*F*_(11,583)_ = 1.84, *p* ≤ 0.05, $\eta_{p}^{2}$ = 0.03].

**FIGURE 4:**
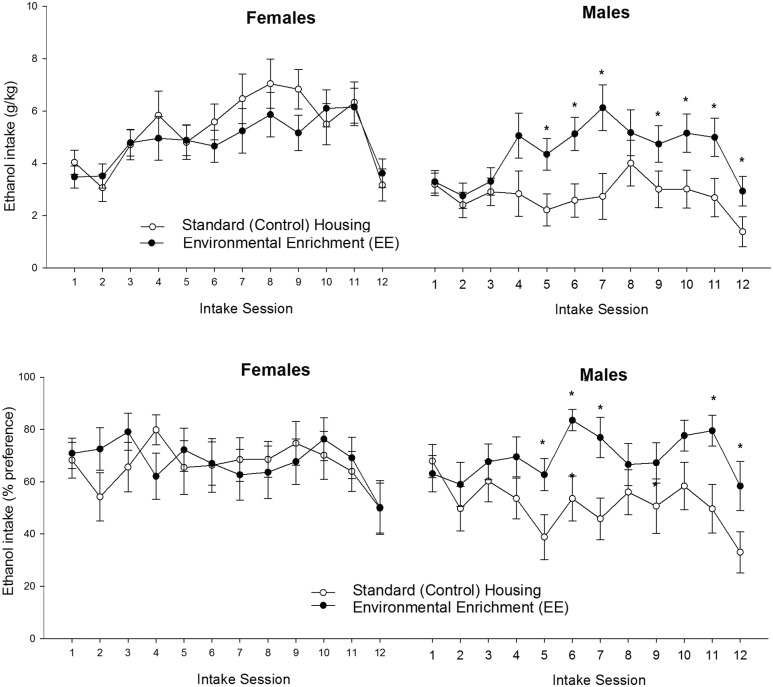
**Ethanol intake (g/kg and percent preference) (**upper and lower panels**, respectively) in male and female Wistar rats as a function of rearing conditions during postnatal days 21 to 42 (standard control housing or environmental enrichment, CTRL and EE groups, respectively) and intake session.** The asterisk sign (^∗^) indicates a significant difference between EE and CTRL male rats, in a given session. The data are expressed as mean ± SEM.

The significant three-way interaction, which is depicted in **Figure 4**, was explored via follow-up ANOVAs (Rearing condition at adolescence × Session) for each sex. The ANOVA conducted for females only revealed a significant main effect of Sessions [*F*_(11,297)_ = 7.37, *p* ≤ 0.001; $\eta_{p}^{2}$ = 0.21]. The *post hoc* tests indicated significantly greater drinking scores on sessions 7–11 than in sessions 1, 2 (*p* ≤ 0.05) or 12 (challenge session, *p* ≤ 0.001). This pattern was not affected by the rearing conditions during infancy or adolescence.

The ANOVA for males, in turn, yielded significant main effects of Session and Rearing conditions at adolescence, [*F*_(11,286)_ = 6.54, *p* ≤ 0.0001, $\eta_{p}^{2}$ = 0.20; *F*_(1,26)_ = 9.75, *p* ≤ 0.005, $\eta_{p}^{2}$ = 0.27; respectively]. The interaction between these factors was significant [*F*_(11,286)_ = 7.64, *p* ≤ 0.0001, $\eta_{p}^{2}$ = 0.11]. Males exposed to EE drank, regardless of whether they had been exposed to MS or not, significantly more than males in the CTRL group, from the second week of testing onward. Among males, the planned comparisons revealed significantly greater ethanol drinking (g/kg) in EE than in CTRL rats at sessions 5, 6, 7, 9, 10, 11 and also during the challenge at session 12 (*p* < 0.005, 0.001, 0.005, 0.05, 0.005, 0.005, and 0.05, respectively).

The analysis of percent ethanol preference yielded similar results to those obtained with absolute intake scores. The ANOVA revealed a significant main effect of Session [*F*_(11,583)_ = 3.95, *p* ≤ 0.0001, $\eta_{p}^{2}$ = 0.07] and a significant interaction between Sex, Session and Rearing conditions at adolescence [*F*_(11.583)_ = 1.87, *p* ≤ 0.05, $\eta_{p}^{2}$ = 0.04]. The follow-up ANOVA for females revealed a lack of significant main effects or significant interactions, whereas the ANOVA for males indicated significant main effects of Session and Rearing conditions at adolescence, as well as a significant interaction between these factors [F_(11,286)_ = 3.10, *p* ≤ 0.0001, $\eta_{p}^{2}$ = 0.11; *F*_(1.26)_ = 4.52, *p* ≤ 0.05, $\eta_{p}^{2}$ = 0.15; *F*_(11,286)_ = 1.92, *p* ≤ 0.05, $\eta_{p}^{2}$ = 0.07, respectively]. The planned comparisons indicated, among males, significantly greater ethanol percent preference in the EE than in the AFR group at sessions 5, 6, 7, 11, and 12 (*p* < 0.05, 0.01, 0.001, 0.05, and 0.05, respectively).

The ANOVA for water consumption scores (ml/100 g of body weight, descriptive data not shown) yielded significant main effects of Sex [*F*_(1,53)=_ 6.38, *p* ≤ 0.05, $\eta_{p}^{2}$ = 0.11] and Session [*F*_(11,583)=_ 8.92, *p* ≤ 0.001, $\eta_{p}^{2}$ = 0.14]. The Session × Sex interaction also achieved significance [*F*_(11.583)=_ 2.78, *p* ≤ 0.005, $\eta_{p}^{2}$ = 0.05]. The planned comparisons indicated that females, irrespective of the rearing conditions experimented during infancy and adolescence, drank significantly more than males, at sessions 3, 8, 11, and 12 (all *p* > 0.05).

Rearing conditions at infancy did not exert a significant main effect, nor were involved in any significant interaction, in any of the variables analyzed.

### Experiment 2

Maternal separation at infancy, as an individual factor, significantly reduced the percent time spent in the open arms of the EPM [*F*_(1,28)__=_ 4.09, *p* ≤ 0.05, $\eta_{p}^{2}$ = 0.13], without altering the total number of arm entries. The ANOVA for the latter variable revealed no significant main effects or significant interactions. The ANOVA for rearing behavior indicated a significant interaction between Rearing conditions at infancy and Rearing conditions at adolescence [*F*_(1,28)__=_ 4.97, *p* ≤ 0.05, $\eta_{p}^{2}$ = 0.17]. The *post hoc* revealed that rearing was significantly greater in the AFR-EE group than in the AFR-CTRL group. Rearing was measured only in the open arms.

**FIGURE 5:**
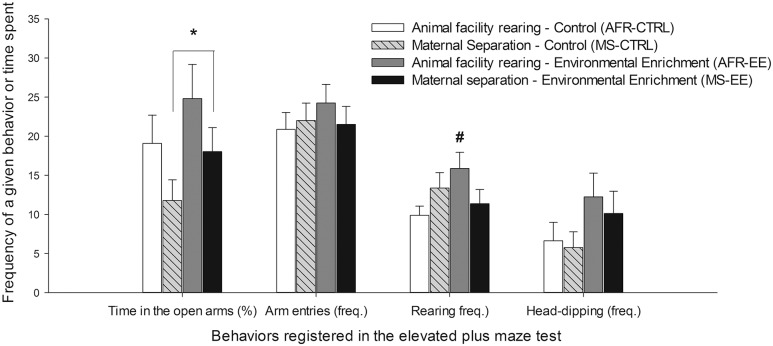
**Time spent in the open arms (%), total number of arm entries and frequency of rearing and head-dipping (expressed as mean ± SEM) in the elevated plus maze (EPM, Experiment 2) test.** The animals, male Wistar rats, were exposed or not to maternal separation on postnatal days (PDs) 1–21 (AFR and MS groups, respectively) and housed under environmental enrichment or standard (control) housing conditions on PDs 21–42 (EE and CTRL groups, respectively). The tests were conducted at PD42. The asterisk sign (^∗^) indicates that maternal separation at infancy, as an individual factor, significantly reduced the percent time spent in the open arms of the EPM. The pound sign (#) indicates that frequency of rearing was significantly greater in the AFR-EE group than in the AFR-CTRL group.

The ANOVA for stretching revealed an interaction between Rearing conditions at infancy, Rearing conditions at adolescence and the section of the EPM where this behavior was measured [*F*_(1,28)__=_ 4.05, *p* ≤ 0.05, $\eta_{p}^{2}$ = 0.13]. The planned comparisons indicated that AFR-EE animals made significantly more stretching in the open arms than the rest of the groups. MS rats exhibited, irrespective of whether or not they had been given EE, significantly more sniffing than AFR animals in the closed arms, but not in the rest of sections, [significant main effect of section: *F*_(2,56)__=_ 40.35, *p* ≤ 0.001, $\eta_{p}^{2}$ = 0.59; significant Section × Rearing conditions at infancy interaction: *F*_(2,56)__=_ 3.88, *p* ≤ 0.05, $\eta_{p}^{2}$ = 0.12]. EE animals shown, irrespective of whether or not they had been given MS, a trend for greater head-dipping [main effect of EE: *F*_(1,28)__=_ 3.71, *p* = 0.06, $\eta_{p}^{2}$ = 0.12]. Head-dipping was only measured in the open arms. **Figure 5** illustrates time spent in the open arms (%), total number of arm entries and frequency of rearing and head-dipping in the EPM, whereas the lower section of **Table 1** presents mean and SEM across conditions, for frequency of stretching (open, closed arms) and sniffing (center, open, closed arms).

Across groups, intake of NaCl (ml/100g) exhibited a three-fold reduction between conditioning (3.69 ± 0.28) and testing (1.13 ± .30). This reduction, suggestive of acquired taste aversion, did not seem to be affected by Rearing conditions at infancy or adolescence. Mean ± SEM consumption of NaCl (ml/100 g) across groups, during conditioning and testing, were as follows: MS-CTRL 3.96 ± 0.46 and 1.31 ± 0.70, AFR-CTRL 3.37 ± 0.30 and 0.63 ± 0.40, MS-EE 3.36 ± 0.63 and 0.70 ± 0.44, AFR-EE 4.05 ± 0.81 and 1.88 ± 0.75. The ANOVA confirmed these impressions. The analysis only revealed a significant main effect of Session [*F*_(1,28)__=_ 74.25, *p* ≤ 0.001, $\eta_{p}^{2}$ = 0.73]. Maternal Separation and EE did not exert a significant main effect upon intake of NaCl nor were involved in any significant interaction. Similarly, the latter factors did not significantly affect ethanol-induced sleep time, latency to lose the righting reflex after the ethanol administration or blood ethanol levels at awakening time. Mean ± SEM for these variables are in **Table 1.**

### Experiment 3

Overall locomotion in the CSF remained unaffected by rearing conditions at infancy or adolescence. The ANOVA for total number of section entries indicated the lack of significant main effects or significant interactions. Mean and SEM across groups were as follows MS-CTRL 104.28 ± 4.95., AFR-CTRL 114.50 ± 14.66., MS-EE 127.71 ± 6.21., AFR-EE 101.2 ± 9.60.

**FIGURE 6:**
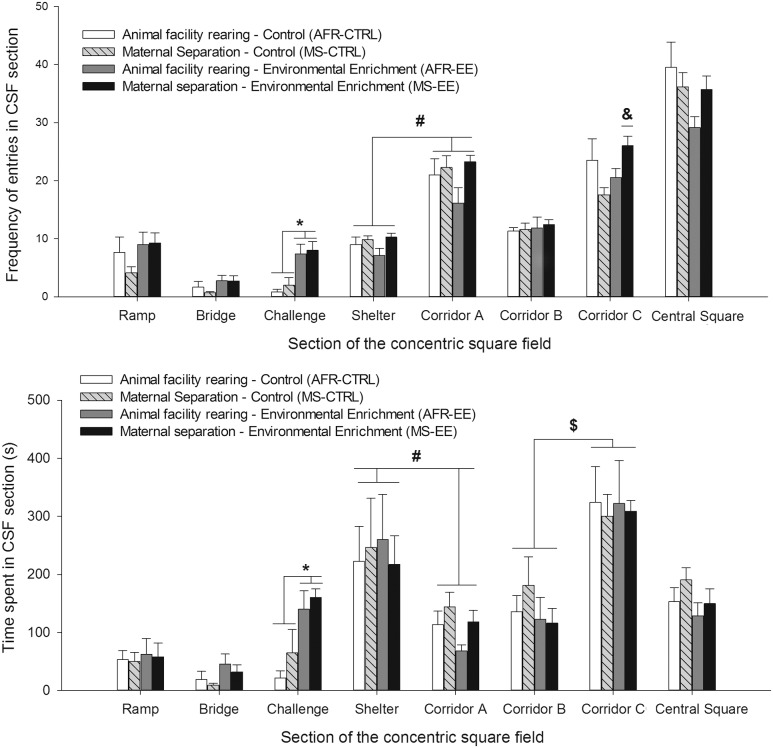
**Frequency of entries and time spent (upper and lower panels, respectively) in each section of the concentric square field, in male Wistar rats tested at postnatal day 42.** The data are expressed as mean ± SEM and shown as a function of rearing conditions during postnatal days 1–21 (animal facility rearing or daily episodes of maternal separation, AFR and MS groups, respectively) and rearing conditions during postnatal days 21–42 [standard (i.e., control) housing or environmental enrichment, CTRL and EE groups, respectively]. The asterisk sign (^∗^) indicates that EE groups exhibited, when compared to animals reared under standard (control) housing conditions (CTRL groups, irrespective of whether they had been given or not maternal separation during infancy), significantly greater time spent and number of entries in the challenge sector. The pound sign (#) indicates that the animals, regardless of the rearing conditions during infancy and adolescence, exhibited significantly greater time spent, and significantly less number of entries, in the sheltered area than in corridor A. These effects were not affected rearing conditions at infancy or adolescence. The ampersand sign (&) indicates that frequency of entries in corridor C was significantly greater in MS-EE animals than in MS-CTRL or AFR-EE counterparts. The currency ($) sign indicates that, regardless the rearing conditions during infancy and adolescence, animals of all groups spent significantly more time in corridor C than in corridor B. The data are expressed as mean ± SEM.

**Figure 6** illustrates mean number of entries and time spent (s) in the risk taking/assessment areas of the apparatus. The ANOVAs indicated, for both variables, a significant main effect of EE and Sector and a significant interaction between these factors, [Number of entries: *F*_(1,24)__=_ 12.23, *p* ≤ 0.05, $\eta_{p}^{2}$ = 0.34; *F*_(2,48)_ = 19.5, *p* < 0.001, $\eta_{p}^{2}$ = 0.45 and *F*_(2,48)_ = 3.61, *p* ≤ 0.05, $\eta_{p}^{2}$ = 0.13, respectively; Time spent: *F*_(1,24)_ = 14.02, *p* ≤ .001, $\eta_{p}^{2}$ = 0.36; *F*_(2,48)_ = 9.66, *p* ≤ 0.001, $\eta_{p}^{2}$ = 0.29 and *F*_(2,48)_ = 5.42, *p* ≤ 0.01, $\eta_{p}^{2}$ = 0.18, respectively]. The planned comparisons indicated that, when compared to CTRL animals (i.e., those given standard housing after the weaning), EE animals exhibited (regardless of the rearing conditions experimented during infancy) significantly greater time spent (*p* < 0.005) and number of entries (*p* < 0.001) in the CHA sector and a trend for greater number of entries an time spent in the BRIDGE (both *p* = 0.07).

The ANOVA for number of entries in the sheltered area and in the corridor A yielded a significant main effect of sector [*F*_(1,24)_ = 239.76, *p* ≤ 0.001, $\eta_{p}^{2}$ = 0.91]. The rats – regardless their rearing conditions at infancy or adolescence – exhibited significantly more number of entries in the corridor than in the sheltered area. The ANOVA also indicated a borderline effect of MS stress, as an individual factor [*F*_(1,24)__=_ 3.69, *p* = 0.06, $\eta_{p}^{2}$ = 0.13]. MS rats (as a group, irrespective of whether they had been exposed to EE or CTRL housing conditions during adolescence) exhibited greater number of entries into these areas than AFR rats, although this difference did not reach statistical significance. Time spent in the sheltered area was significantly greater than time spent in corridor A [*F*_(1,24)_ = 11.44, *p* ≤ .005, $\eta_{p}^{2}$ = 0.32], an effect that was not significantly affected by Rearing conditions at infancy stress or at adolescence.

The CHA sector can be accessed via corridor B or corridor C, yet only corridor C allows entry into the brighter RAMP, which in turn leads to the bridge. The RM ANOVA for number of entries in these corridors yielded a significant main effect of sector [*F*_(1,24)_ = 81.16, *p* ≤ 0.0001, $\eta_{p}^{2}$ = 0.77]. The interactions between MS stress and Enrichment [*F*_(1,24)_ = 4.75, *p* ≤ 0.05, $\eta_{p}^{2}$ = 0.17] and between MS Stress, Enrichment and Sector [*F*_(1,24)_ = 6.15, *p* ≤ 0.05, $\eta_{p}^{2}$ = 0.20] also achieved significance. The *post hoc* revealed that the number of entries into corridor B was similar between the different groups. The *post hoc* also indicated that the number of entries into corridor C was significantly greater in rats from group MS-EE than in rats from group MS-CTRL (*p* < 0.05). The ANOVA for time spent in these corridors only indicated that animals, regardless their rearing conditions during infancy or adolescence, spent significantly more time in C than in B [*F*_(1,24)_ = 25.98, *p* ≤ 0.001, $\eta_{p}^{2}$ = 0.52]. These results area illustrated in **Figure 6.**

The ANOVAs for time spent and frequency of entries into the central square (see **Figure 6**), and for nose-poke into the CHA holes (data not shown) did not yield significant main effects or interactions.

## Discussion

The main finding of the present study was that exposure to EE throughout adolescence induced a significant increase in ethanol intake and preference during periadolescence and young adulthood.

Environmental enrichment male, but not female, rats exhibited a two-fold increase in ethanol intake when compared to counterparts given standard housing, and achieved up to 80% ethanol predilection vs. water. The sex-related difference can simply be due to the fact that female rats (Lancaster et al., 1996; Doremus et al., 2005) or mice (Lopez and Laber, 2015) often exhibit increased ethanol intake and preference than males, which in turn can impede assessment of treatments that increase ethanol predilection. In other words, it is possible that females in the present study exhibited a ceiling effect in terms of ethanol intake or preference. An important finding was that the differences in ethanol intake between EE and control males were still significant in the last testing day, when ethanol concentration was increased from 5 to 7.5%.

In the present study, the rats were exposed (or not) to daily episodes of MS (duration: 180 min), throughout infancy. The decision of using 180 min of MS, instead of 15 or 360 min, was based on a previous study (Kawakami et al., 2007) that reported faster development of ethanol-induced behavioral sensitization after MS180, but not after MS15. Behavioral sensitization is the gradual increment in the motor-stimulating effects of ethanol following repeated ethanol administration, thought to reflect the transition from controlled to problematic ethanol drinking (Camarini and Pautassi, 2016).

Chronic MS, unlike EE, did not significantly affect ethanol intake in the present study. This was not unexpected, as the few previous studies that tested ethanol intake shortly after termination of MS reported no effect of MS upon ethanol drinking at infancy, adolescence-periadolescence or early adulthood. Rats exposed to MS180 or AFR during PD1-13 exhibited no differences in ethanol intake (6%, tested via intraoral infusions Pautassi et al., 2012) at PD15. Other studies used the more conventional exposure to MS360 on PD1-21. Palm et al. (2013) reported no differences in adolescent ethanol intake between MS360 and AFR controls (only males were employed). In a second study, Daoura et al. (2011) assessed ethanol intake via an intermittent, three-bottle, test (0.0, 5, or 20% ethanol), for 5 weeks, starting on PD26 (adolescence) or PD68 (adulthood). Ethanol intake, which again was assessed in males only, was exacerbated in animals exposed to MS360, yet only when testing began at adulthood. Similarly, Roman et al. (2003) assessed ethanol intake in the ethanol-preferring AA strain from late adolescence - early adulthood (i.e., approximately PD77) to full adulthood (i.e., until PD120). Early maternal separation did not significantly affect ethanol intake in females. Intriguingly, the male’s acceptance of ethanol was unaffected by early rearing conditions during the first 5 weeks of testing, yet after that – when the rats were in full adulthood – the MS360 group exhibited a significant increase in ethanol drinking.

Taken together, these studies (i.e., Roman et al., 2003; Daoura et al., 2011; Pautassi et al., 2012; Palm et al., 2013) and the results obtained in the present study cement the notion that, in rats, the effects of MS upon ethanol intake remain silent during infancy or adolescence and are expressed only when subjects reach full adulthood. It should be noted that it is not the case that MS was devoid of effects in the present study. Maternal separation induced significantly less exploration of the open spaces of the EMP and resulted in greater time spent in the sheltered area (and in the corridor leading to it) of the CSF. These results are consistent with the hypothesis that MS increases anxiety responses (Huot et al., 2001), yet it seems that the magnitude of this change was not substantial enough to influence ethanol drinking or, as indicated earlier, it is possible that the anxiety phenotype only affects ethanol intake at adulthood. This may be a consequence of ethanol intake being driven by different neurobiological mechanisms in adolescent vs. adults. Adolescent, but not adult, rats exhibit conditioned place preference by ethanol (Philpot et al., 2003; Pautassi et al., 2008), a result suggestive of greater ethanol-induced appetitive effects in the younger animals. Ethanol drinking in adolescents may be driven by these appetitive effects of ethanol; whereas the anti-anxiety effects of ethanol could be more involved in drinking during adulthood. Consistent with this postulate, Samson et al. (1998) suggested that rats require protracted experience with ethanol drinking to learn about ethanol’s anti-anxiety effects. Also noteworthy is that maternal separation affects a plethora of neural systems, yet the most prominent is the heightened responsiveness of the hypothalamic–pituitary–adrenal system toward subsequent stressors (Pryce et al., 2002). In Huot et al. (2001), for instance, adult rats exposed during infancy to maternal separation drank more ethanol and exhibited greater corticosterone response to airpuff startle, than non-stressed controls.

Environmental enrichment has been proposed as a non-pharmacological tool to reduce drug-induced adaptive changes (Solinas et al., 2009; de Carvalho et al., 2010). Swiss mice housed during adolescence in large-than-usual cages equipped with house-like objects, a running wheel and several tubes, were resistant to the development of ethanol-induced behavioral sensitization (Rueda et al., 2012), a behavioral proxy for the neural changes taking place during the transition from regular drug use to addiction (Camarini and Pautassi, 2016). Yet other studies provided contradictory information. Early work (Rockman et al., 1986, 1989) found greater ethanol intake after EE, although these researchers only tested ethanol intake in adulthood and after lengthy (i.e., ≥90 days) EE exposure. A facilitating effect of EE upon drug reactivity has also been observed with other drugs. EE exposure resulted in heightened amphetamine (Bowling and Bardo, 1994) or nicotine (Ewin et al., 2015) induced conditioned place preference.

What are the mechanisms that, in the present study, led to increased ethanol intake after EE? We analyzed, in male rats exposed to EE, sensitivity to the hypnotic-sedative effects and to the post-ingestive, aversive effects of ethanol. These effects have been suggested to serve as barriers that prevent initiation or escalation into ethanol intake, and differences in these effects have been used to explain differences in ethanol intake between adolescent and adult rats (Spear and Varlinskaya, 2010; Spear and Swartzwelder, 2014). Our hypothesis was that EE rats would be resistant to these effects, yet this was not corroborated. Ethanol induced significant flavor aversion and readily resulted in hypnosis, yet these effects were fairly similar across rearing conditions. Significant limitations of Experiment 2 were, however, the use of a single dose of ethanol and the lack of vehicle or unpaired controls in the taste conditioning procedure. This introduces the possibility that the aversion to the salty solution obeys to the lingering, toxic effects of ethanol, and perhaps differentially so across groups. Another important limitation of this study is that the animals were individually housed during the course of each ethanol intake test, and in-between tests they were again pair-housed. This repeated social isolation probably resulted in significant stress and, therefore, should be considered a factor contributing to the observed effects.

A study (Fernández-Teruel et al., 2002) found greater head-dipping and ethanol intake after EE, in a rat strain exhibiting high anxiety and low levels of novelty seeking. This points to the possibility that, in the present study, EE facilitated ethanol intake by increasing novelty-seeking. Evidence supporting this is that EE rats exhibited greater head-dipping and rearing behaviors in the EMP test, as well as more stretching in the open arm and in the center of the apparatus, than their non-enriched counterparts. Previous work suggest that head-dipping and rearing reflect novelty seeking and exploration (Fernández-Teruel et al., 2002; Lever et al., 2006), whereas stretching involves risk assessment (Bailey and Crawley, 2009). Enriched animals, although only those also exposed to MS stress, also exhibit significantly greater number of transfers and time spent in the bright compartment of the LDB, when compared to the rest of the groups. This finding represents a priming effect of MS on subsequent EE exposure, indicating that these two environmental treatments can sometimes act in an additive fashion. Perhaps more important, EE significantly increased frequency of entries and time spent in the challenge area, a risk-taking area of the CSF. The access to this area required jumping through an elevated hole. The brightly lit, open and elevated bridge was also more visited by enriched than by non-enriched rats, irrespective of the rearing conditions experimented during infancy, although this was a trend that did not achieve statistical significance. The overall number of entries in the different sections of the CSF was unaffected by EE, indicating that EE effects upon risk-taking behavior were not a by-product of unspecific changes in motor activity.

Previous studies indicate that the changes that define an enriched homecage, relative to the conditions of the standard housing, do not have to be dramatic to alter ethanol’s effects or intake. Lopez and Laber (2015) found increased ethanol consumption at adulthood after chronic single housing during adolescence. This effect was inhibited by simply adding cotton nestlets to the homecage during the isolation period. The EE in the present study, on the other hand, involved a significantly larger homecage featuring new configurations of objects that kept rotating and the possibility to perform physical activity. This raises the possibility that EE effects upon ethanol intake depend on the magnitude of the stimulation provided by the environment: relatively low magnitudes of EE may inhibit ethanol intake, yet exposure to a relatively high-magnitude EE treatment may result in greater ethanol intake. Similar complex relationships have been claimed to explain the controversial effects of stress upon ethanol intake. Studies have found greater (Caplan and Puglisi, 1986), diminished (Boyce-Rustay et al., 2008), or unaltered (Ponce et al., 2004) ethanol intake after stress exposure, and some claim that these apparent disparate results could be explained by the Yerkes–Dobson law (reviewed in Miczek et al., 2008), with low stress inducing behavioral activation and promoting ethanol intake whereas high stress inducing behavioral depression and a reduced ethanol intake. Under this framework, the EE rats in this study may have perceived the EE treatment as a mild stressor. Consistent with this, it has been suggested that rats exposed to EE exhibit a mild stress-like phenotype, which may inoculate from subsequent response to more intense stressors (Crofton et al., 2015). These are, of course, just hypotheses and more work should be done to describe the effects of EE and its underlying mechanisms. It is noteworthy, however, that compared to adults, adolescents have been described to be more reactive to stress and to stress-ethanol interactions. In a recent study we found significantly greater ethanol intake time in adolescent, but not in adult, rats given chronic restraint stress (Fernandez et al., 2016).

In summary, the present study confirmed that the effects of MS stress upon ethanol intake are not expressed during late adolescence, in spite of MS inducing other behavioral changes indicative of heightened anxiety response. Perhaps more important, animals that had been exposed to EE throughout adolescence subsequently exhibited significantly greater ethanol intake, an effect found in males only and not affected by MS. The promoting effect of EE upon ethanol intake was not related to changes in the aversive or sedative effects of ethanol, nor in ethanol’s metabolism. Instead, it seems that EE heightened exploration of novel stimuli and risk-taking behaviors in the CSF test. Further studies should assess if EE may affect ethanol intake and preference via alterations in novelty-seeking and risk-taking behaviors.

## Author Contributions

LB run all the experiments, supervised the construction of the mazes, processed the data and wrote an initial draft of the MS. RP had the original research idea, designed the studies, supervised the running of the Experiments, conducted the statistical analyses, graphed the data and wrote the final version of the paper. MF help design the experiments along with RMP, run all the experiments, supervised the construction of the mazes and help wrote the initial draft of the paper. All authors revised the final version of the paper.

## Conflict of Interest Statement

The authors declare that the research was conducted in the absence of any commercial or financial relationships that could be construed as a potential conflict of interest.

## References

[B1] Acevedo M. B. Molina J. C. Nizhnikov M. E. Spear N. E. Pautassi R. M. (2010). High ethanol dose during early adolescence induces locomotor activation and increases subsequent ethanol intake during late adolescence. *Dev. Psychobiol.* 52 424–440. 10.1002/dev.20444 20373327 PMC3050506

[B2] Arias C. Pautassi R. M. Molina J. C. Spear N. E. (2010). A comparison between taste avoidance and conditioned disgust reactions induced by ethanol and lithium chloride in preweanling rats. *Dev. Psychobiol.* 52 545–557. 10.1002/dev.20460 20806327 PMC3056390

[B3] Arnold J. L. Siviy S. M. (2002). Effects of neonatal handling and maternal separation on rough-and-tumble play in the rat. *Dev. Psychobiol.* 41 205–215. 10.1002/dev.10069 12325135

[B4] Bailey K. R. Crawley J. N. (2009). “Anxiety-related behaviors in mice,” in *Methods of Behavior Analysis in Neuroscience*, 2nd Edn, ed. Buccafusco J. J. (Boca Raton, FL: CRC Press/Taylor & Francis).

[B5] Bowling S. L. Bardo M. T. (1994). Locomotor and rewarding effects of amphetamine in enriched, social, and isolate reared rats. *Pharmacol. Biochem. Behav.* 48 459–464. 10.1016/0091-3057(94)90553-3 8090815

[B6] Boyce-Rustay J. M. Janos A. L. Holmes A. (2008). Effects of chronic swim stress on EtOH-related behaviors in C57BL/6J, DBA/2J and BALB/cByJ mice. *Behav. Brain Res.* 186 133–137. 10.1016/j.bbr.2007.07.031 17822784 PMC2695676

[B7] Camarini R. Pautassi R. M. (2016). Behavioral sensitization to ethanol: neural basis and factors that influence its acquisition and expression. *Brain Res. Bull.* 125 53–78. 10.1016/j.brainresbull.2016.04.006 27093941

[B8] Caplan M. A. Puglisi K. (1986). Stress and conflict conditions leading to and maintaining voluntary alcohol consumption in rats. *Pharmacol. Biochem. Behav.* 24 271–280. 10.1016/0091-3057(86)90350-3 3952116

[B9] Crofton E. J. Zhang Y. Green T. A. (2015). Inoculation stress hypothesis of environmental enrichment. *Neurosci. Biobehav. Rev.* 49 19–31. 10.1016/j.neubiorev.2014.11.017 25449533 PMC4305384

[B10] Cruz F. C. Quadros I. M. Planeta Cda S. Miczek K. A. (2008). Maternal separation stress in male mice: long-term increases in alcohol intake. *Psychopharmacology (Berl.)* 201 459–468. 10.1007/s00213-008-1307-4 18766329 PMC4367178

[B11] Daoura L. Haaker J. Nylander I. (2011). Early environmental factors differentially affect voluntary ethanol consumption in adolescent and adult male rats. *Alcohol. Clin. Exp. Res.* 35 506–515. 10.1111/j.1530-0277.2010.01367.x 21143247

[B12] Dawson D. A. Grant B. F. Li T. K. (2007). Impact of age at first drink on stress-reactive drinking. *Alcohol. Clin. Exp. Res.* 31 69–77. 10.1111/j.1530-0277.2006.00265.x 17207104

[B13] de Carvalho C. R. Pandolfo P. Pamplona F. A. Takahashi R. N. (2010). Environmental enrichment reduces the impact of novelty and motivational properties of ethanol in spontaneously hypertensive rats. *Behav. Brain Res.* 208 231–236. 10.1016/j.bbr.2009.11.043 19962407

[B14] DeWit D. J. Adlaf E. M. Offord D. R. Ogborne A. C. (2000). Age at first alcohol use: a risk factor for the development of alcohol disorders. *Am. J. Psychiatry* 157 745–750. 10.1176/appi.ajp.157.5.745 10784467

[B15] Doremus T. L. Brunell S. C. Rajendran P. Spear L. P. (2005). Factors influencing elevated ethanol consumption in adolescent relative to adult rats. *Alcohol. Clin. Exp. Res.* 29 1796–1808. 10.1097/01.alc.0000183007.65998.aa 16269909

[B16] Dyr W. Wyszogrodzka E. Paterak J. Siwinska-Ziolkowska A. Malkowska A. Polak P. (2016). Ethanol-induced conditioned taste aversion in Warsaw Alcohol High-Preferring (WHP) and Warsaw Alcohol Low-Preferring (WLP) rats. *Alcohol* 51 63–69. 10.1016/j.alcohol.2015.11.011 26992702

[B17] Enoch M. A. (2012). The influence of gene-environment interactions on the development of alcoholism and drug dependence. *Curr. Psychiatry Rep.* 14 150–158. 10.1007/s11920-011-0252-9 22367454 PMC3470472

[B18] Ewin S. E. Kangiser M. M. Stairs D. J. (2015). The effects of environmental enrichment on nicotine condition place preference in male rats. *Exp. Clin. Psychopharmacol.* 23 387–394. 10.1037/pha0000024 26167715

[B19] Fabio M. C. Macchione A. F. Nizhnikov M. E. Pautassi R. M. (2015). Prenatal ethanol increases ethanol intake throughout adolescence, alters ethanol-mediated aversive learning, and affects mu but not delta or kappa opioid receptor mRNA expression. *Eur. J. Neurosci.* 41 1569–1579. 10.1111/ejn.12913 25865037

[B20] Fernandez M. Fabio M. C. Nizhnikov M. E. Spear N. E. Abate P. Pautassi R. M. (2014). Maternal isolation during the first two postnatal weeks affects novelty-induced responses and sensitivity to ethanol-induced locomotor activity during infancy. *Dev. Psychobiol.* 56 1070–1082. 10.1002/dev.21192 24374748

[B21] Fernandez M. S. Fabio M. C. Miranda-Morales R. S. Virgolini M. B. De Giovanni L. N. Hansen C. (2016). Age-related effects of chronic restraint stress on ethanol drinking, ethanol-induced sedation, and on basal and stress-induced anxiety response. *Alcohol* 51 89–100. 10.1016/j.alcohol.2015.11.009 26830848 PMC5123306

[B22] Fernández-Teruel A. Driscoll P. Gil L. Aguilar R. Tobena A. Escorihuela R. M. (2002). Enduring effects of environmental enrichment on novelty seeking, saccharin and ethanol intake in two rat lines (RHA/Verh and RLA/Verh) differing in incentive-seeking behavior. *Pharmacol. Biochem. Behav.* 73 225–231. 10.1016/S0091-3057(02)00784-0 12076741

[B23] Francis D. D. Kuhar M. J. (2008). Frequency of maternal licking and grooming correlates negatively with vulnerability to cocaine and alcohol use in rats. *Pharmacol. Biochem. Behav.* 90 497–500. 10.1016/j.pbb.2008.04.012 18508115 PMC2559944

[B24] Gruber E. DiClemente R. J. Anderson M. M. Lodico M. (1996). Early drinking onset and its association with alcohol use and problem behavior in late adolescence. *Prev. Med.* 25 293–300. 10.1006/pmed.1996.0059 8781007

[B25] Huot R. L. Thrivikraman K. V. Meaney M. J. Plotsky P. M. (2001). Development of adult ethanol preference and anxiety as a consequence of neonatal maternal separation in Long Evans rats and reversal with antidepressant treatment. *Psychopharmacology (Berl.)* 158 366–373. 10.1007/s002130100701 11797057

[B26] Karlsson O. Roman E. (2016). Dose-dependent effects of alcohol administration on behavioral profiles in the MCSF test. *Alcohol* 50 51–56. 10.1016/j.alcohol.2015.10.003 26695588

[B27] Kawakami S. E. Quadros I. M. Takahashi S. Suchecki D. (2007). Long maternal separation accelerates behavioural sensitization to ethanol in female, but not in male mice. *Behav. Brain Res.* 184 109–116. 10.1016/j.bbr.2007.06.023 17675171

[B28] Lancaster F. E. Brown T. D. Coker K. L. Elliott J. A. Wren S. B. (1996). Sex differences in alcohol preference and drinking patterns emerge during the early postpubertal period. *Alcohol. Clin. Exp. Res.* 20 1043–1049. 10.1111/j.1530-0277.1996.tb01945.x 8892526

[B29] Lever C. Burton S. O’Keefe J. (2006). Rearing on hind legs, environmental novelty, and the hippocampal formation. *Rev. Neurosci.* 17 111–133. 10.1515/REVNEURO.2006.17.1-2.111 16703946

[B30] Lopez M. F. Laber K. (2015). Impact of social isolation and enriched environment during adolescence on voluntary ethanol intake and anxiety in C57BL/6J mice. *Physiol. Behav.* 148 151–156. 10.1016/j.physbeh.2014.11.012 25446196 PMC4425642

[B31] Meyerson B. J. Augustsson H. Berg M. Roman E. (2006). The Concentric Square Field: a multivariate test arena for analysis of explorative strategies. *Behav. Brain Res.* 168 100–113. 10.1016/j.bbr.2005.10.020 16356558

[B32] Miczek K. A. Yap J. J. Covington H. E. (2008). Social stress, therapeutics and drug abuse: preclinical models of escalated and depressed intake. *Pharmacol. Ther.* 120 102–128. 10.1016/j.pharmthera.2008.07.006 18789966 PMC2713609

[B33] Middleton F. A. Varlinskaya E. I. Mooney S. M. (2012). Molecular substrates of social avoidance seen following prenatal ethanol exposure and its reversal by social enrichment. *Dev. Neurosci.* 34 115–128. 10.1159/000337858 22572756 PMC3560533

[B34] National-Research-Council (1996). *Guide for the Care and Use of Laboratory Animals.* Washington, DC: National Academy Press.

[B35] Ottoni E. B. (2000). EthoLog 2.2: a tool for the transcription and timing of behavior observation sessions. *Behav. Res. Methods Instrum. Comput.* 32 446–449. 10.3758/BF03200814 11029818

[B36] Palm S. Daoura L. Roman E. Nylander I. (2013). Effects of rearing conditions on behaviour and endogenous opioids in rats with alcohol access during adolescence. *PLoS ONE* 8:e76591 10.1371/journal.pone.0076591 PMC3788749 24098535

[B37] Pautassi R. M. Myers M. Spear L. P. Molina J. C. Spear N. E. (2008). Adolescent but not adult rats exhibit ethanol-mediated appetitive second-order conditioning. *Alcohol. Clin. Exp. Res.* 32 2016–2027. 10.1111/j.1530-0277.2008.00789.x 18782343 PMC2588482

[B38] Pautassi R. M. Nizhnikov M. E. Fabio M. C. Spear N. E. (2012). Early maternal separation affects ethanol-induced conditioning in a nor-BNI insensitive manner, but does not alter ethanol-induced locomotor activity. *Pharmacol. Biochem. Behav.* 100 630–638. 10.1016/j.pbb.2011.11.005 22108648 PMC3256748

[B39] Philpot R. M. Badanich K. A. Kirstein C. L. (2003). Place conditioning: age-related changes in the rewarding and aversive effects of alcohol. *Alcohol. Clin. Exp. Res.* 27 593–599. 10.1111/j.1530-0277.2003.tb04395.x 12711921

[B40] Pilatti A. Godoy J. C. Brussino S. Pautassi R. M. (2013a). Underage drinking: prevalence and risk factors associated with drinking experiences among Argentinean children. *Alcohol* 47 323–331. 10.1016/j.alcohol.2013.02.001 23591270

[B41] Pilatti A. Godoy J. C. Brussino S. A. Pautassi R. M. (2013b). Patterns of substance use among Argentinean adolescents and analysis of the effect of age at first alcohol use on substance use behaviors. *Addict. Behav.* 38 2847–2850. 10.1016/j.addbeh.2013.08.007 24018229

[B42] Ponce L. F. Pautassi R. M. Spear N. E. Molina J. C. (2004). Nursing from an ethanol-intoxicated dam induces short- and long-term disruptions in motor performance and enhances later self-administration of the drug. *Alcohol. Clin. Exp. Res.* 28 1039–1050. 10.1097/01.alc.0000131298.32045.96 15252290

[B43] Pryce C. R. Ruedi-Bettschen D. Dettling A. C. Feldon J. (2002). Early life stress: long-term physiological impact in rodents and primates. *News Physiol. Sci.* 17 150–155. 12136043 10.1152/nips.01367.2001

[B44] Rockman G. E. Borowski T. B. Glavin G. B. (1986). The effects of environmental enrichment on voluntary ethanol consumption and stress ulcer formation in rats. *Alcohol* 3 299–302. 10.1016/0741-8329(86)90005-4 3778645

[B45] Rockman G. E. Gibson J. E. Benarroch A. (1989). Effects of environmental enrichment on voluntary ethanol intake in rats. *Pharmacol. Biochem. Behav.* 34 487–490. 10.1016/0091-3057(89)90545-5 2623006

[B46] Roman E. Hyytia P. Nylander I. (2003). Maternal separation alters acquisition of ethanol intake in male ethanol-preferring AA rats. *Alcohol. Clin. Exp. Res.* 27 31–37. 10.1097/01.ALC.0000047352.88145.80 12544002

[B47] Roman E. Stewart R. B. Bertholomey M. L. Jensen M. L. Colombo G. Hyytia P. (2012). Behavioral profiling of multiple pairs of rats selectively bred for high and low alcohol intake using the MCSF test. *Addict. Biol.* 17 33–46. 10.1111/j.1369-1600.2011.00327.x 21521426 PMC5472351

[B48] Rothman E. F. Edwards E. M. Heeren T. Hingson R. W. (2008). Adverse childhood experiences predict earlier age of drinking onset: results from a representative US sample of current or former drinkers. *Pediatrics* 122 e298–e304. 10.1542/peds.2007-3412 18676515

[B49] Rueda A. V. Teixeira A. M. Yonamine M. Camarini R. (2012). Environmental enrichment blocks ethanol-induced locomotor sensitization and decreases BDNF levels in the prefrontal cortex in mice. *Addict. Biol.* 17 736–745. 10.1111/j.1369-1600.2011.00408.x 22126132

[B50] Samson H. H. Slawecki C. J. Sharpe A. L. Chappell A. (1998). Appetitive and consummatory behaviors in the control of ethanol consumption: a measure of ethanol seeking behavior. *Alcohol. Clin. Exp. Res.* 22 1783–1787. 10.1111/j.1530-0277.1998.tb03980.x 9835295

[B51] Schneider T. Turczak J. Przewlocki R. (2006). Environmental enrichment reverses behavioral alterations in rats prenatally exposed to valproic acid: issues for a therapeutic approach in autism. *Neuropsychopharmacology* 31 36–46. 10.1038/sj.npp.1300767 15920505

[B52] Solinas M. Thiriet N. El Rawas R. Lardeux V. Jaber M. (2009). Environmental enrichment during early stages of life reduces the behavioral, neurochemical, and molecular effects of cocaine. *Neuropsychopharmacology* 34 1102–1111. 10.1038/npp.2008.51 18463628

[B53] Spanagel R. Montkowski A. Allingham K. Stohr T. Shoaib M. Holsboer F. (1995). Anxiety: a potential predictor of vulnerability to the initiation of ethanol self-administration in rats. *Psychopharmacology (Berl)* 122 369–373. 10.1007/BF02246268 8657835

[B54] Spear L. P. (2000). The adolescent brain and age-related behavioral manifestations. *Neurosci. Biobehav. Rev.* 24 417–463. 10.1016/S0149-7634(00)00014-2 10817843

[B55] Spear L. P. Swartzwelder H. S. (2014). Adolescent alcohol exposure and persistence of adolescent-typical phenotypes into adulthood: a mini-review. *Neurosci. Biobehav. Rev.* 45C, 1–8. 10.1016/j.neubiorev.2014.04.012 PMC4134704 24813805

[B56] Spear L. P. Varlinskaya E. I. (2010). Sensitivity to ethanol and other hedonic stimuli in an animal model of adolescence: implications for prevention science? *Dev. Psychobiol.* 52 236–243. 10.1002/dev.20457 20222058 PMC3045082

[B57] Winer B. Brown D. R. Michels K. M. (1991). *Statistical Principles in Experimental Design.* New York, NY: McGraw Hill.

